# Associations of objectively measured total duration and maximum bout length of standing at work with lower-extremity pain intensity: a 2-year follow-up of construction and healthcare workers

**DOI:** 10.1186/s12891-020-03868-0

**Published:** 2021-01-07

**Authors:** Lars-Kristian Lunde, Suzanne Merkus, Markus Koch, Stein Knardahl, Morten Wærsted, Kaj Bo Veiersted

**Affiliations:** National Institute of Occupational Health, P.O. Box 5330, Majorstuen, 0304 Oslo, Norway

**Keywords:** Accelerometers, Construction work, Healthcare work, Lower-extremity pain, Musculoskeletal disorders, Objective measures, Standing, Physical work exposures, Prospective design

## Abstract

**Background:**

Musculoskeletal disorders are among the major reasons for years lived with disability. Approximately one third of the European working population report lower-extremity discomfort and many attribute these discomforts to work-related factors. Employees in the healthcare and construction sectors reports high levels of lower-extremity pain and commonly relate the pain to their profession. These workers spend a large part of their workday standing. Periods of prolonged standing is suggested to increase lower-extremity symptoms, but this cannot be concluded on, since limited evidence is available from longitudinal studies using objective measures. This study aimed to determine possible associations between objectively measured total duration and maximum bout length of static- and dynamic standing at work and lower-extremity pain intensity (LEPi) among Norwegian construction- and healthcare workers.

**Methods:**

One-hundred and twenty-three construction and healthcare workers wore two accelerometers for up to four consecutive days, to establish standing behavior at baseline. The participants reported LEPi (Likert scale 0–9) for the preceding 4 weeks at baseline and after 6, 12, 18, and 24 months. We investigated associations between standing at work and average and change in LEPi using linear mixed models with significance level *p* ≤ 0.05.

**Results:**

Total duration of static- and dynamic standing showed weak associations with average LEPi, for the total sample and for construction workers. Maximum bout of static- and dynamic standing was associated with average LEPi in construction workers, but not in healthcare workers. Furthermore, we found no associations between standing and change in LEPi over the 2-year follow-up in any of our analyses.

**Conclusions:**

This study indicate that objectively measured standing is associated with average LEPi over 2-years follow-up in construction workers, and that maximal bout of standing have a stronger association to LEPi than total duration. For every 10 min added to the maximal length of continuous standing during an average workday, we found approximately one unit increase in pain on a 0–9 scale. The lack of significant findings in analyses on healthcare workers suggest that the association between standing and LEPi depend on work-tasks, gender and/or other sector-specific factors.

**Supplementary Information:**

The online version contains supplementary material available at 10.1186/s12891-020-03868-0.

## Background

Musculoskeletal disorders are among the major reasons for years lived with disability. Approximately 30% of the European working population report lower-extremity discomfort [1–3], and the prevalence of work-related lower-extremity pain (LEP) for these workers is estimated to be 16% [3]. In the Norwegian working population in 2016, 29% reported LEP and 1/3 of these attributed their pain to work-related factors [4].

Healthcare and construction employees report high levels of LEP and commonly relate the pain to their profession [2, 4, 5]. These workers also spend the majority of their workday in upright positions [6, 7]. Periods of prolonged standing is suggested to increase lower-extremity symptoms [2, 8, 9], with most common presented mechanisms being muscle fatigue and increased blood pooling [8, 10, 11].

However, the association between standing and lower-extremity pain cannot be concluded on, since limited evidence is available from longitudinal studies using objective measures [9]. Self-reported exposures are vulnerable to recall bias, question interpretation and participants’ pain level, resulting in attenuated validity. Thus, we should strive to implement objective measurements in investigations of physical exposures [9, 12], and preferably measure several consecutive days, to better capture variations in exposure between workdays [13, 14].

Few studies have measured standing exposure objectively for consecutive days to investigate its association to LEP, and to our knowledge, there are no such studies with longitudinal design. Two recent cross-sectional studies with objective measures of blue-collar and office workers weakly indicated that lower-extremity symptoms increased with more standing [15, 16]. Valid knowledge on the association between standing and LEP is crucial to guide preventive approaches by policy makers, practitioners, and workplaces aiming to reduce lower-extremity pain caused by mechanical exposures.

Musculoskeletal disorders is commonly of multifactorial origin and work related factors like squatting/kneeling [17], lifting [18, 19] and psychological and social work factors [20] could be of importance when studying the relationship between standing and LEP. Individual factors like age [21, 22], gender [22], smoking [23] and high body mass index [22, 24] may also affect this relationship and act as confounders.

This study was designed as a part of a larger prospective cohort study [25]. In the present study, we aim to determine the association between objectively measured standing at work and lower-extremity pain intensity (LEPi) in construction- and healthcare workers over a 2-year period. Pain reporting was chosen as outcome due to its clinical significance, its scientific properties and relevance, and due to its common use and comprehensibility in the general population. We considered two plausible scenarios: the measured exposure lead to a change in pain over time, or the measured exposure is connected to the maintained level of pain over time.

We tested four hypotheses concerning the association between standing and LEPi during a 2-years follow-up with two different approaches to pain (1-2 and 3-4):

1. Total duration of standing at work is associated with the average LEPi.
2. Maximum bout duration of standing at work is associated with the average LEPi.
3. Total duration of standing at work is associated with change in LEPi between baseline and four follow-up time points.
4. Maximum bout duration of standing at work is associated with change in LEPi between baseline and four follow-up time points.

## Methods

### Study population and design

The target population was employees in four construction companies and two local healthcare distributors in the Oslo area (total: *n* = 1165; construction workers: *n* = 580; healthcare workers: *n* = 585). We presented the purpose, format, and methods of the study at informational meetings located at the potential participants work site. Five hundred and ninety-four participants (construction workers: *n* = 293; healthcare workers: *n* = 301) agreed to participate and data collection started in the 1st quarter of 2014. At baseline, all participants answered the study questionnaire. Of the 594 participants, 178 construction workers and 193 healthcare workers additionally agreed to participate in technical measurements at baseline, and of these we selected 66 construction and 72 healthcare workers based on logistics (availability, work schedules and profession). These measurements consisted of the assessment of standing by two accelerometers worn 24 h a day, for up to four consecutive days while maintaining a short diary. In the diary, the participants were instructed to note the time of day they got out of bed in the morning, when they started their workday (if workday), when they ended their workday (if workday), and when they went to bed at night, for the days they were measured. We followed up all participants by questionnaires every 6 months for a total of 2 years, with data collection ending in the 1st quarter of 2017. Please see Lunde et al. for the timeline established for the larger prospective cohort study the present study was designed as a part of [25]. We excluded participants with inadequate skills in reading and writing Norwegian. In the group with technical measurements, we had the additional exclusion criteria of being pregnant, having a known allergic reaction to plaster, tape, or bandages.

### Instrumentation for technical measurements

To measure the acceleration, position, and angle of body segments, we used ActiGraph GT3X+ sensors (ActiGraph LLC, Pensacola, Florida, United States) with a sampling frequency of 30 Hz. Following a standardized set-up, one accelerometer was placed on the participant’s right thigh (medially between the iliac crest and the upper crest of the patella), and one was placed on the back, leveled with T1-T2 [25–27]. The accelerometers are lightweight (19 g) and were fixed on the skin using double-sided tape (Fixomull, BSN Medical, Hamburg, Germany) and covered with transparent film (Tegaderm, 3 M, St. Paul, Minnesota, United States).

### Standing during work

Participants wore accelerometers for up to four continuous days at baseline, and from their diaries, we identified periods of work. We excluded periods where the accelerometers were not worn, or periods where measurement data criteria were unsatisfactory (shorter than 4 h or 75% of the mean length of all respective periods) [28]. This was implemented as an effort to increase data quality, in terms of increasing the possibility of exposure data being representative for a full “normal workday”. I.e. a very short period of measurement within a working day are more prone to be affected by the variation in exposure throughout that day. We present data on standing as the average total daily duration for measured workdays (based on aggregated minutes of standing each workday) and the average maximum standing bout (based on longest continuous, uninterrupted period of standing each workday) in minutes. Calculations were done with a custom-made MATLAB-based program, Acti4 (NRCWE, Denmark and BAuA, Germany). The Actigraph GT3X+ sensors setup used in this study is found to be valid for detecting durations of standing during free living [26, 27].

#### Differentiation between static and dynamic standing

We differentiated between static standing, where the participants stood completely still, and dynamic standing, which in addition to static standing also included situations of standing position with small movements, but without regular walking. For a detailed description we refer to the study by Skotte et al., where the algorithms for stand (what we here call static standing) and move (static standing + move is what we in the present study refer to as dynamic standing) are explained in full [27].

#### Self-reported standing at work

As a proxy for potential change in exposure during the 2-year follow-up, participants reported the fraction of their daily work performed standing, with the response alternatives (0) *Never*, (1) *Very small part of the time*, (2) *Approximately 25% of the time,* (3) *Approximately 50% of the time,* (4) *Approximately 75% of the time,* and (5) *Almost all the time* [29].

### Lower-extremity pain intensity

Subjects rated their pain intensity in hip, knee, and feet/ankle (pain in either or both sides, with no differentiation between left and right) during the preceding 4 weeks, on a four-point scale (*not troubled* = 0, *a little troubled* = 1, *rather intensely troubled* = 2 and *very intensely troubled* = 3) [30]. A mannequin drawing facilitated localization of body regions. The reported pain levels were summed to create a LEPi score (0–9). E.g. if a participant reported to be very intensely troubled (= 3) in all of the regions hip, knee, and feet/ankle, this would sum up to a LEPi of 9. A participant that was not troubled (= 0) in any region would get a LEPi of 0. In cases where subjects had reported only on one or two out of the three pain sites for a specific follow-up, we aggregated the available data.

### Covariates

#### Individual factors

We collected information on age, gender, seniority in profession, BMI (kg/m^2^), and smoking status by questionnaire. Participants were classified as smokers if they smoked daily or occasionally.

#### Self-reported manual handling

Participants were asked to estimate the frequency of lifting something weighing more than 20 kg during regular workdays, with the response alternatives (0) *No*, (1) *Yes, 1–4 times*, (2) *yes, 5–19 times* and (3) *yes, at least 20 times a day* [29].

#### Self-reported kneeling

Participants reported the fraction of a regular workday performed kneeling, with response alternatives (0) *Never*, (1) *Very small part of the time*, (2) *Approximately 25% of the time,* (3) *Approximately 50% of the time,* (4) *Approximately 75% of the time,* and (5) *Almost all the time* [29].

#### Psychosocial factors

The current study assessed decision control, fair and empowering leadership, and social climate using items taken from the General Nordic Questionnaire for Psychological and Social Factors at work (QPSNordic), a validated instrument for research and tool for employers to monitor and improve working conditions [31, 32]. For each of the four subjects a mean was calculated based on the responses.

### Statistical analyses

We tested associations between standing and LEPi with linear mixed models fitted by restricted maximum likelihood with a random intercept added for subject. In separate analyses, total duration (in minutes) of standing at work and maximum bout duration (in minutes) of standing at work were treated as main exposure variable, while LEPi was treated as the dependent variable. We tested the pre-set hypotheses with two different approaches. The first approach tested whether the total duration and the maximal bout duration were associated with the AVERAGE LEPi during the 2-year follow-up. Here, standing was included as main effect and we assumed that the time effect of the exposure on the outcome was equal for all time points. For this approach, we removed baseline pain observations, aiming to reduce bias from a revers causal effect between lower-extremity pain and standing at baseline. The second approach tested whether the total duration of standing and the maximal bout duration of standing were associated with the CHANGE in LEPi (ΔLEPi) from baseline to the four time points during follow-up. Here, we included standing as main effect, time as categorical variable, and an interaction between standing and time (standing*time). We treated time as a categorical variable to provide estimates for every follow-up. For both approaches, we performed analyses on the total sample and stratified by work sector. Confounding variables were pre-selected and checked for co-linearity (seniority was excluded due to high correlation with age). Four models were developed for each separate analysis: Model 1) crude association between objectively measured total duration or maximal bout of standing (static and dynamic) and LEPi (average or change); Model 2) as model 1 + adjustments for age, gender, smoking and BMI; Model 3) as model 2 + adjustments for heavy lifting and kneeling; and Model 4) as model 3 + adjustments for social climate, decision control, fair leadership, and empowering leadership. We additionally carried out supplementary analyses on associations between the total duration of on-feet activity (dynamic standing + walking) and the average and change in LEPi. Further, we investigated potential differences between responders and non-responders on follow-up questionnaires and between objective measurement group and questionnaire only group by testing group variables with Independent sample t-test and Wilcoxon rank-sum test. As an indication of stability in work characteristics over the 2 years of follow-up, we tested possible changes in self-reported standing between baseline, 6-, 12-, 18-, and 24 months with Friedman’s analysis of variance.

We conducted statistical analyses in STATA, version 15.1 (StataCorp, College Station, TX, USA) and associations were calculated per 100 min for total duration of standing and per 10 min for maximal bout duration of standing, with 95% confidence intervals.

## Results

**Fig. 1:**
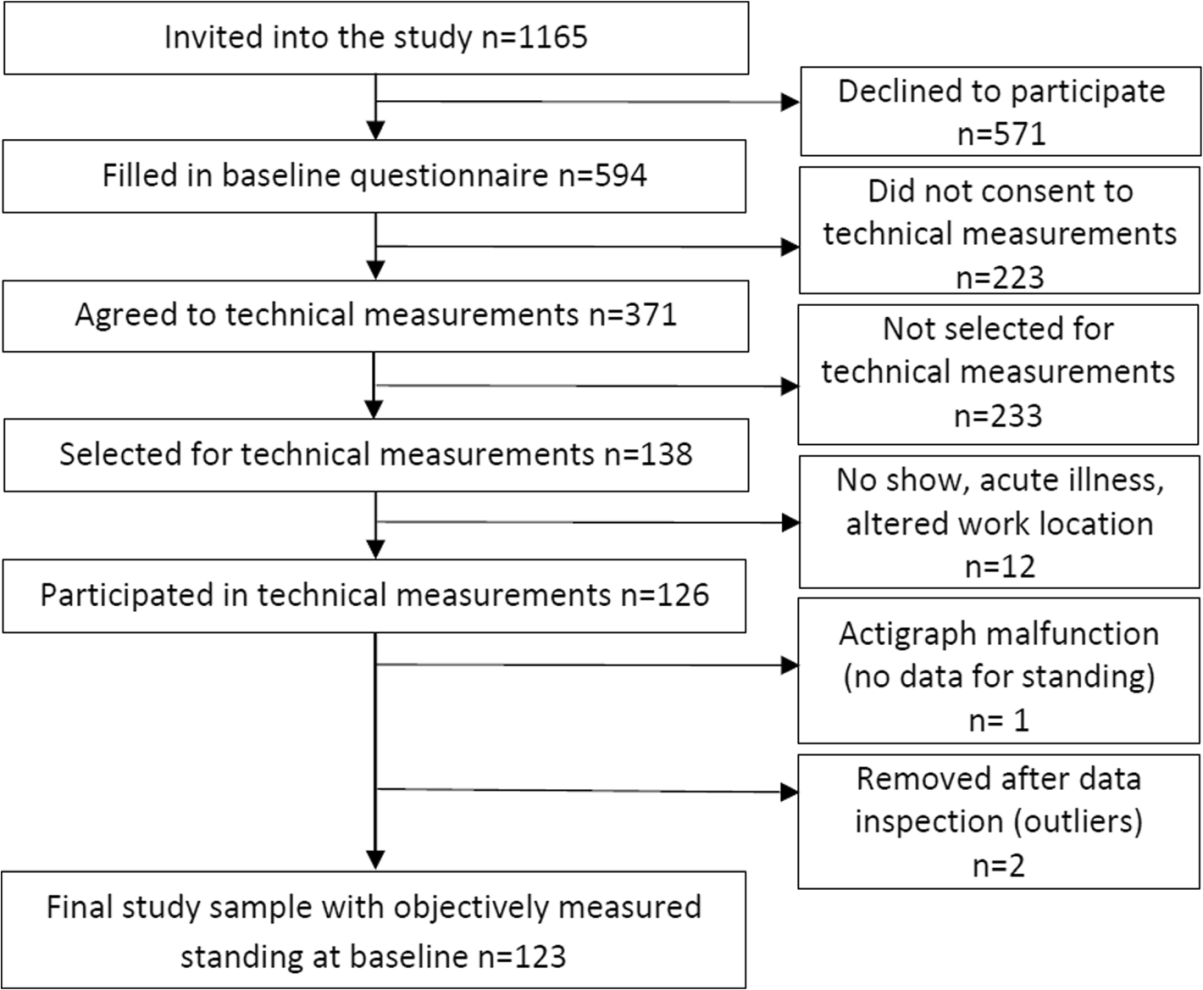
Participant flow

Of the 138 selected employees, twelve did not participate due to not showing up at the agreed time of measurement, acute illness or change in work location. Due to accelerometer malfunction, one person had no data on standing exposure. Further, we removed two persons considered to be outliers from statistical analyses (unnaturally low standing values including a large deviation from self-reported standing for a normal day). Thus, the final study sample consisted of 123 employees (construction *n* = 61; healthcare *n* = 62). See Table 1 for subject characteristics and Fig. 1 for participant flow diagram. Self-reported standing at baseline differed significantly between the study sample with technical measures and the group only answering questionnaires (*p* = 0.030), where the technical group reported higher levels. We found no differences in self-reported standing (Construction *p* = 0.496; Healthcare *p* = 0.474) between baseline, 6, 12, 18, and 24 months.

**Table 1:** Descriptive characteristics of the study participants at baseline (*n* = 123)

Variables	Total (*n =* 123)			Construction (*n* = 61)			Healthcare (*n* = 62)		
	%	mean	SD	%	mean	SD	%	mean	SD
Age (years)	∙	42.3	11.9	∙	39.9	13.6	∙	44.7	9.5
Gender (male)	60.2	∙	∙	98.4	∙	∙	22.6	∙	∙
Body mass index (kg/m^2^)	∙	25.4	3.6	∙	25.7	3.3	∙	25.1	3.8
Smokers	29.3	∙	∙	31.1	∙	∙	27.4	∙	∙
Seniority in profession (years)	∙	16.7	11.3	∙	17.1	12.8	∙	16.3	9.7
Normal work hours per week	∙	36.8	4.3	∙	37.8	4.0	∙	35.7	4.2
Work hours measured per day	∙	7.6	1.5	∙	8.2	1.8	∙	7.1	0.8
Total duration static standing at work (min)	∙	140.8	65.3	∙	156.8	69.4	∙	125.0	57.3
Total duration dynamic standing at work (min)	∙	220.1	95.3	∙	249.0	102.4	∙	191.8	78.7
Max bout static standing at work (min)	∙	7.0	4.0	∙	7.3	4.1	∙	6.6	3.8
Max bout dynamic standing at work (min)	∙	13.7	8.1	∙	14.2	8.7	∙	13.3	7.4
Heavy lifting at work (0–3)^a^	∙	1.0	1.1	∙	1.5	1.3	∙	0.4	0.6
Kneeling at work (0–5)^b^	∙	1.3	1.3	∙	1.6	1.4	∙	1.0	1.1
Social climate at work (1–5)^c^	∙	4.0	0.7	∙	4.0	0.7	∙	4.0	0.7
Decision control at work (1–5)^d^	∙	3.1	0.7	∙	3.1	0.6	∙	3.0	0.8
Fair leadership (1–5)^d^	∙	4.0	0.8	∙	4.0	0.7	∙	4.0	0.9
Empowering leadership (1–5)^d^	∙	3.5	1.0	∙	3.1	0.9	∙	3.9	1.0
Lower extremity pain intensity score (0–9)^e^	∙	1.5	1.9	∙	1.2	1.7	∙	1.7	2.0

^a^Response alternatives: (0) No, (1) Yes, 1–4 times, (2) yes, 5–19 times and (3) yes, at least 20 times a day

^b^Response alternatives: (0) Never, (1) Very small part of the time, (2) Approximately 25% of the time, (3) Approximately 50% of the time, (4) Approximately 75% of the time, and (5) Almost all the time

^c^Response alternatives for supportive, trustful and comfortable climate: (1) very little or not at all, (2) rather little, (3) somewhat, (4) rather much and (5) very much

^d^Response alternatives: (1) very seldom or never, (2) rather seldom, (3) sometimes, (4) rather often and (5) very often or always

^e^Response alternatives: (0) not troubled, (1) a little troubled, (2) rather intensely troubled, (3) very intensely troubled - aggregated for pain sites hip, knee and feet/ankle

### Missing data

There was 123 subjects with valid measurements for at least one workday, while 101 had valid data for two or more workdays. Fifty-three subjects responded to all questionnaires, while 70 failed to respond to one or more follow-up questionnaires. Twenty-three, 41, 41, and 48% of the subjects did not respond to the questionnaire at 6, 12, 18, and 24 months or missed the questions necessary to create a LEPi-score at the respective follow-up. In the construction sector, there was no statistically significant differences in age, gender, objectively measured standing at work, or baseline LEPi between those who only answered the baseline questionnaire and those who additionally responded to one or more follow-up questionnaires. In the healthcare sector, the follow-up non-responders were younger, and consisted of more males.

### Average lower-extremity pain intensity

#### Total duration of static standing

Analyses of all workers showed statistically significant and positive associations between total duration of static standing and average pain during follow-up for models 2 and 3. The crude and fully adjusted models (1 and 4) showed similar trends, but had smaller effect sizes and more uncertainty in estimates. For the construction sector, models 1–3 returned significant estimates. Analyses on healthcare workers showed no associations between static standing and average LEPi during follow-up (Table 2).

**Table 2:** Associations between total duration of static and dynamic standing (per 100 min) and average LEPi during follow-up (Approach 1)

	Model 1			Model 2			Model 3			Model 4		
	Observations = 303/154/149			Observations = 297/153/144			Observations = 296/152/144			Observations = 293/151/142		
	Coef.	95% CI	*P*-value	Coef.	95% CI	*P-*value	Coef.	95% CI	*P-*value	Coef.	95% CI	*P-*value
**Static standing**												
All workers	0.46	−0.03,0.94	0.064	0.58	0.07,1.09	**0.027**	0.52	0.002,1.04	**0.049**	0.48	−0.06,1.02	0.079
Construction	0.63	0.05,1.21	**0.033**	0.68	0.10,1.26	**0.022**	0.63	0.01,1.25	**0.045**	0.52	−0.10,1.14	0.101
Healthcare	0.41	−0.49,1.31	0.369	0.37	−0.58,1.31	0.445	0.33	−0.58,1.24	0.478	0.22	−0.74,1.18	0.650
**Dynamic standing**												
All workers	0.33	0.01,0.65	**0.046**	0.43	0.08,0.74	**0.017**	0.38	0.02,0.75	**0.040**	0.36	−0.01,0.73	0.056
Construction	0.38	0.00,0.76	**0.050**	0.42	0.03,0.80	**0.033**	0.42	−0.002,0.83	0.051	0.29	−0.16,0.74	0.205
Healthcare	0.46	−0.18,1.11	0.156	0.45	−0.23,1.13	0.196	0.40	−0.27,1.06	0.242	0.27	−0.42,0.96	0.266

Observations: total observations included in linear mixed models for all workers/construction/healthcare

Dependent variable: the average pain intensity from 6 months to 24 months follow-up (T2, T3, T4, T5)

Independent variables:

Model 1: Static standing and dynamic standing (minutes per workday)

Model 2: As model 1 + Age, Gender, Smoking, BMI

Model 3: As model 2 + Heavy lifting, Kneeling

Model 4: As model 3 + Social climate, Decision control, Fair leadership, Empowering leadership

#### Total duration of dynamic standing

Analyses of associations between total duration of dynamic standing and average pain on all workers showed similar trends as for static standing, with models 1–3 being statistically significant. Separated analyses on construction workers showed significant results for models 1 and 2, while analyses on healthcare showed no associations (Table 2).

#### Maximum bouts of static standing

Analyses on all workers showed no associations between maximum bouts of static standing and average LEPi during follow-up. For construction workers, all models were statistically significant and had positive associations, while associations for healthcare workers were negative and non-significant (Table 3).

**Table 3:** Associations between maximum bouts of static and dynamic standing (per 10 min) and average LEPi during follow-up (Approach 1)

	Model 1			Model 2			Model 3			Model 4		
	Observations = 303/154/149			Observations = 297/153/144			Observations = 296/152/144			Observations = 293/151/142		
	Coef.	95% CI	*P-*value	Coef.	95% CI	*P-*value	Coef.	95% CI	*P-*value	Coef.	95% CI	*P-*value
**Static standing**												
All workers	0.30	−0.57,1.17	0.500	0.29	−0.60,1.17	0.524	0.33	−0.54,1.20	0.451	0.35	−0.53,1.23	0.437
Construction	1.50	0.40,2.59	**0.007**	1.21	0.09,2.32	**0.034**	1.14	0.02,2.27	**0.047**	1.23	0.12,2.33	**0.030**
Healthcare	−0.70	−1.99,0.60	0.292	−0.80	−2.18,0.58	0.258	− 0.70	−2.05,0.65	0.310	−0.77	−2.20,0.66	0.292
**Dynamic standing**												
All workers	0.58	0.12,1.05	**0.014**	0.58	0.11,1.05	**0.016**	0.57	0.10,1.10	**0.017**	0.55	0.07,1.02	**0.023**
Construction	1.13	0.47,1.79	**0.001**	1.02	0.37,1.68	**0.002**	1.00	0.33,1.67	**0.004**	1.17	0.52,1.82	**0.001**
Healthcare	0.25	−0.41,0.91	0.458	0.21	− 0.51,0.92	0.570	0.25	−0.44,0.94	0.485	0.11	−0.61,0.83	0.761

Observations: total observations included in linear mixed models for all workers/construction/healthcare

Dependent variable: the average pain intensity from 6 months to 24 months follow-up (T2, T3, T4, T5)

Independent variables:

Model 1: Maximum bout with static or dynamic standing (minutes per work day)

Model 2: As model 1 + Age, Gender, Smoking, BMI

Model 3: As model 2 + Heavy lifting, Kneeling

Model 4: As model 3 + Social climate, Decision control, Fair leadership, Empowering leadership

#### Maximum bouts of dynamic standing

For maximum bouts of dynamic standing, analyses on all workers and construction workers only, showed significant positive associations with average pain for all models. Analyses on healthcare workers showed non-significant associations with positive estimates (Table 3).

### Change in lower-extremity pain intensity

#### Total duration of static standing

Analyses of all workers together and construction- and healthcare workers separately, showed no statistically significant associations between total duration of static standing at work and change in LEPi during follow-up (Table 4). See supplementary Table A for all models.

**Table 4:** Associations between total duration of static and dynamic standing at work (per 100 min) and change in LEPi during follow-up Approach 2)

		Static standing						Dynamic standing					
		Model 1			Model 4			Model 1			Model 4		
		Observations = 426/215/211			Observations = 412/212/200			Observations = 426/215/211			Observations = 412/212/200		
		Coef.	95% CI	*P-*value	Coef.	95% CI	*P-*value	Coef.	95% CI	*P-*value	Coef.	95% CI	*P-*value
Total	T2	−0.03	− 0.46,0.40	0.896	− 0.07	− 0.50,0.37	0.761	0.02	− 0.28,0.31	0.920	0.003	−0.29,0.30	0.987
	T3	0.02	−0.48,0.53	0.926	0.03	−0.49,0.54	0.921	0.08	−0.24,0.40	0.609	0.09	−0.24,0.42	0.580
	T4	−0.33	−0.80,0.15	0.177	−0.33	− 0.84,0.17	0.197	− 0.04	−0.45,0.17	0.383	−0.13	− 0.45,0.20	0.440
	T5	0.10	−0.42,0.61	0.714	0.002	−0.52,0.53	0.995	0.14	−0.19,0.49	0.399	0.08	−0.27,0.43	0.648
Construction	T2	−0.07	−0.57,0.44	0.789	−0.12	− 0.63,0.39	0.651	− 0.05	− 0.38,0.28	0.766	− 0.09	− 0.43,0.25	0.604
	T3	0.24	−0.38,0.86	0.444	0.16	−0.48,0.80	0.617	0.19	−0.19,0.56	0.331	0.14	−0.25,0.53	0.490
	T4	− 0.35	− 0.91,0.21	0.223	−0.34	− 0.97,0.30	0.297	− 0.13	− 0.48,0.23	0.490	− 0.12	− 0.50,0.26	0.535
	T5	0.25	−0.34,0.83	0.410	0.25	−0.36,0.85	0.426	0.14	−0.24,0.52	0.482	0.14	−0.25,0.52	0.485
Healthcare	T2	− 0.05	−0.82,0.71	0.894	− 0.16	− 0.93,0.62	0.695	0.08	−0.48,0.63	0.786	0.03	−0.54,0.60	0.928
	T3	−0.44	−1.31,0.42	0.315	−0.44	−1.32,0.44	0.330	−0.28	− 0.92,0.36	0.385	− 0.25	− 0.91,0.41	0.458
	T4	−0.40	−1.22,0.43	0.347	−0.52	−1.37,0.33	0.232	−0.23	− 0.84,0.38	0.458	− 0.26	− 0.89,0.37	0.420
	T5	−0.59	−1.53,0.34	0.213	−0.85	−1.81,0.11	0.082	−0.32	−1.04,0.39	0.377	−0.54	−1.29,0.20	0.154

T2: 6 months, T3: 12 months, T4: 18 months, T5: 24 months; Observations: total observations included in models for total/construction/healthcare; *P*-values ≤0.05 in bold

Dependent variable: Change in pain between T1 and TX. Independent variables: Model 1: Static standing or dynamic standing at work (minutes per work day), Model 4: As model 1 + Age, Gender, BMI, Smoking, Heavy lifting, Kneeling, Social climate, Decision control, Fair leadership, Empowering leadership

Tables including all models (Model 2 and Model 3) are shown in supplementary Tables A and B

#### Total duration of dynamic standing

For total duration of dynamic standing we found no significant associations with change in LEPi for any of our analyses (Table 4). See supplementary Table B for all models.

#### Maximal bouts of static standing

When analyzing associations between maximal bouts of static standing at work and change in LEPi during follow-up we found no statistically significant results (Table 5). See supplementary Table C for all models.

**Table 5:** Associations between maximum bouts of static and dynamic standing at work (per 10 min) and change in LEPi during follow-up (Approach 2)

		Static standing						Dynamic standing					
		Model 1			Model 4			Model 1			Model 4		
		Observations = 426/215/211			Observations = 412/212/200			Observations = 426/215/211			Observations = 412/212/200		
		Coef.	95% CI	*P-*value	Coef.	95% CI	*P-*value	Coef.	95% CI	*P-*value	Coef.	95% CI	*P-*value
Total	T2	− 0.25	− 1.01,0.50	0.508	− 0.23	− 0.99,0.53	0.550	0.05	−0.35,0.45	0.810	0.02	−0.38,0.42	0.929
	T3	−0.09	−0.93,0.74	0.828	−0.16	−1.03,0.69	0.714	−0.10	− 0.65,0.45	0.725	− 0.12	−0.67,0.43	0.666
	T4	−0.31	−1.14,0.53	0.470	−0.46	−1.36,0.44	0.311	−0.09	−0.59,0.40	0.710	−0.17	− 0.67,0.33	0.510
	T5	−0.15	−1.00,0.70	0.729	−0.17	−1.03,0.70	0.697	−0.27	−0.86,0.31	0.357	−0.35	− 0.94,0.23	0.236
Construction	T2	−0.07	−1.06,0.93	0.895	−0.06	−1.05,0.94	0.911	0.24	−0.35,0.82	0.428	0.24	−0.34,0.83	0.414
	T3	0.36	−0.69,1.42	0.501	0.34	−0.71,1.40	0.526	0.32	−0.45,1.06	0.427	0.33	−0.44,1.09	0.404
	T4	0.42	−0.62,1.45	0.431	0.37	−0.77,1.52	0.525	−0.19	−1.03,0.64	0.650	−0.36	−1.26,0.53	0.427
	T5	0.29	−0.72,1.31	0.574	0.43	−0.62,1.48	0.426	−0.16	−0.95,0.63	0.690	−0.16	− 0.96,0.65	0.706
Healthcare	T2	−0.44	−1.53,0.66	0.436	−0.51	−1.66,0.64	0.384	−0.09	−0.64,0.47	0.762	−0.15	− 0.71,0.41	0.596
	T3	−0.62	−1.90,0.65	0.340	−0.82	−2.15,0.52	0.229	−0.43	−1.22,0.36	0.284	−0.46	−1.25,0.34	0.262
	T4	−1.06	− 2.36,0.24	0.110	−1.29	− 2.64,0.06	0.060	−0.11	− 0.75,0.53	0.731	− 0.19	− 0.85,0.47	0.574
	T5	−0.88	− 2.24,0.49	0.208	−1.10	−2.50,0.30	0.122	−0.35	−1.20,0.49	0.410	−0.47	−1.33,0.40	0.292

T2: 6 months, T3: 12 months, T4: 18 months, T5: 24 months; Observations: total observations included in models for total/construction/healthcare; *P-*values ≤0.05 in bold

Dependent variable: Change in pain between T1 and TX. Independent variables: Model 1: Maximum bout with static or dynamic standing at work (minutes per work day), Model 4: As model 1 + Age, Gender, BMI, Smoking, Heavy lifting, Kneeling, Social climate, Decision control, Fair leadership, Empowering leadership

Tables including all models (Model 2 and Model 3) are shown in supplementary Tables C and D

#### Maximal bouts of dynamic standing

Analyses using maximal bouts of dynamic standing showed no significant associations with change in LEPi during follow-up (Table 5). See supplementary Tables D for all models.

### Supplementary analyses

In analyses on all workers, on-feet activity (dynamic standing + walking) and average pain showed similar trends as for dynamic standing, with all models being statistically significant with positive estimates. Analyses separated by work sector showed non-significant positive associations. There were no associations between on-feet activity and change in LEPi. See supplementary Tables E and F.

## Discussion

We found weak, positive associations between the total duration of static and dynamic standing at work and average LEPi for all workers and for construction workers only. However, these associations attenuated in models adjusting for work-related psychosocial factors. Analyses using maximal bout of standing for construction workers showed consistent significant results for all models, suggesting that longer bouts are associated with higher average levels of LEPi for these workers. We found no association between standing at work and average LEPi for healthcare workers, and no associations in any of our analyses on standing at work and change in LEPi during follow-up.

There are few studies investigating the association between objectively measured standing and LEPi in free-living individuals [9]. In two cross-sectional studies using objective measures, researchers found weak indications of increased LEP with increased standing duration [15, 16]. Our results did also indicate a weak association between total duration of standing and the average LEPi in construction workers, but this association turned insignificant in models adjusting for work-related psychosocial factors. However, the associations we found between maximal bout of standing and average level of LEPi showed a consistent significant association for all models. On the contrary, the study by Locks found that short bouts of standing throughout the day was associated with increased pain in the knee and hip. However, this may partly be explained by reverse causality in the cross-sectional study design, where those with pain endured only short bouts of static standing [15]. The study by Coenen et al. did not reveal any findings of significance when using usual length standing bouts as exposure [16].

A recent review of laboratory studies, suggested an association between prolonged standing and lower-extremity symptoms, with mechanisms explained through muscle, posture, and blood pooling [8]. Thus, it is reasonable to believe that a prolonged period of static standing increase pain/discomfort, at least temporary. Still, forced static standing with no movement at all, like in laboratory-based studies, is a somewhat artificial work situation, since most workers have some possibility for small movements within or from the static posture. As stated by the review authors, there is little information on dynamic types of standing, which according to mechanistic theories possibly could be more beneficial towards lower-extremity pain by increasing venous return, and offset some of the blood pooling by dynamic muscle action. Still, we found a more prominent association for dynamic compared to static standing for construction workers (Table 3).

Our results diverged between sectors, and the construction sector mainly drove associations found in the total study sample. Such diversions have also been found previously when investigating other mechanical exposures and musculoskeletal outcomes in the same population [33, 34]. Thus, it is plausible that differences in gender and/or work characteristics influence pathogenic mechanisms differentially. In the present population, differences in biomechanical exposure, attitude towards pain, and variation over time is possible explanations for diverging estimates. This may for instance, be a result of differences between the two sectors in the type work performed while standing. There is also likely that the healthcare sector has a more stable exposure level/work situation over time, since they typically work in one building/department for years. Construction workers are on the contrary, connected to projects, which repeatedly change location and environment. Generally, it is plausible that large unspecific groups including various non-related occupations may hide associations in sub-groups.

We found associations between standing and average LEPi, but no associations with change in LEPi during follow-up. This may be related to the population’s relatively high seniority in the profession and that their biomechanical work exposures are not something new suddenly triggering a systematic change in pain. Other explanations could be that a 2-year follow-up is too short to assess changes in pain as a result of standing at work, or that participants reported artificially high pain levels at baseline [30].

For maximal bout of dynamic standing among construction workers, we consider the effect sizes to be reasonable. Coefficients indicate approximately one unit increase in pain on a 0–9 scale with every 10 min added on the average length of standing during a workday (Table 3). Depending on analytic methods, population, baseline status, and type of disorder, a change in patient-reported outcome of 14.5–33% is found to be a minimal clinical important difference for hip, knee, foot and ankle disorders [35]. Thus, a change in the average maximal bout of standing to create this magnitude of pain alteration is within reason.

### Strengths and limitations

The major contribution from this study is the use of validated objective measures for several consecutive days, combined with a 2-year longitudinal design. Another strength are the work sector specific analyses, suggesting a modifying effect of sector.

We did not consider variation between postures, which could be of importance for the relationship between standing and the development of LEPi. Additionally, we did not collect long-term history of LEPi, a variable possibly connected to future LEPi. The comprehensive set up of objective measures and higher level of participant involvement may have led to a more motivated group of workers for the technical measures. The underlying reason for this motivation can only be speculated on. However, for bias to occur the selection must be a function of/affected by both exposure and outcome, something we did not find indications of in this study. Finally, we cannot rule out that a larger sample would reflect findings for smaller effect sizes.

Studying pain-free workers at the start of their carrier, with multiple exposure and outcome assessments for a significant follow-up period, would be important aspects in a study design to handle the uncertainty with work exposure variation, pain fluctuations and the possibility of inverse relationships between exposure and outcome. Further, since most existing theories explain the cause of pain from a temporary/short-term perspective, we would benefit from studies attempting to explain the development of severe and persistent pain that could influence exit from work.

## Conclusions

Objectively measured maximal bout of standing duration during the workday was associated with average levels of lower-extremity pain for construction workers, while results from total duration of standing were less clear. We did not find any associations for healthcare workers, and found no associations between standing behavior and change in LEPi over a 2-year period for any work sector. This study indicate that the associations between standing at work and lower-extremity pain may vary depending on type of work tasks, gender, or sector-specific factors.

Thus, is a potential risk that prolonged standing may cause lower-extremity pain. However, in this relationship there is a potential difference between work sectors. This should be accounted for in future research and in preventive approaches by policy makers, practitioners, and workplaces aiming to reduce lower-extremity pain caused by mechanical exposures.

## Supplementary Information

**Additional file 1: Supplementary A**. Associations between total duration of static standing at work (per 100 min) and change in LEPi during follow-up (Approach 2). **Supplementary B**. Associations between total duration of dynamic standing at work (per 100 min) and change in LEPi during follow-up (Approach 2). **Supplementary C**. Associations between maximum bouts of static standing at work (per 10 min) and change in LEPi during follow-up (Approach 2**). Supplementary D.** Associations between maximum bouts of dynamic standing at work (per 10 min) and change in LEPi during follow-up (Approach 2). **Supplementary E**. Associations between total duration of on-feet activity (per 100 min) and average LEPi during follow-up (Approach 1). **Supplementary F**. Associations between total duration of on-feet activity at work (per 100 min) and change in LEPi during follow-up (Approach 2).

## Data Availability

Data will be available from project leader Kaj Bo Veiersted on reasonable request.

## Abbreviations

LEP: Lower-extremity pain
LEPi: Lower-extremity pain intensity
