# Supplementary material for: Associations of objectively measured total duration and maximum bout length of standing at work with lower-extremity pain intensity: a 2-year follow-up of construction and healthcare workers
Source: BMC Musculoskelet Disord. 2021 Jan 7;22:43. doi: 10.1186/s12891-020-03868-0 (PMC7791765; doi:10.1186/s12891-020-03868-0)
Supplement: Supplementary file 1 — Additional file 1: Supplementary A. Associations between total duration of static standing at work (per 100 min) and change in LEPi during follow-up (Approach 2). Supplementary B. Associations between total duration of dynamic standing at work (per 100 min) and change in LEPi during follow-up (Approach 2). Supplementary C. Associations between maximum bouts of static standing at work (per 10 min) and change in LEPi during follow-up (Approach 2). Supplementary D. Associations between maximum bouts of dynamic standing at work (per 10 min) and change in LEPi during follow-up (Approach 2). Supplementary E. Associations between total duration of on-feet activity (per 100 min) and average LEPi during follow-up (Approach 1). Supplementary F. Associations between total duration of on-feet activity at work (per 100 min) and change in LEPi during follow-up (Approach 2). [file 12891_2020_3868_MOESM1_ESM.docx]

**Supplementary A. Associations between total duration of static standing at work (per 100 minutes) and change in LEPi during follow-up (Approach 2).**

|  |  | Model 1 | | | Model 2 | | | Model 3 | | | Model 4 | | |
| --- | --- | --- | --- | --- | --- | --- | --- | --- | --- | --- | --- | --- | --- |
|  |  | Observations = 426/215/211 | | | Observations = 418/214/204 | | | Observations = 417/213/204 | | | Observations = 412/212/200 | | |
|  |  | Coef. | 95% CI | P-value | Coef. | 95% CI | P-value | Coef. | 95% CI | P-value | Coef. | 95% CI | P-value |
| Total | T2 | -0.029 | -0.461,0.403 | 0.896 | -0.041 | -0.476,0.393 | 0.851 | -0.042 | -0.478,0.395 | 0.851 | -0.067 | -0.501,0.366 | 0.761 |
|  | T3 | 0.024 | -0.478,0.525 | 0.926 | 0.064 | -0.445,0.575 | 0.804 | 0.060 | -0.454,0.574 | 0.819 | 0.026 | -0.489,0.541 | 0.921 |
|  | T4 | -0.327 | -0.803,0.148 | 0.177 | -0.307 | -0.810,0.195 | 0.231 | -0.301 | -0.807,0.205 | 0.244 | -0.332 | -0.836,0.172 | 0.197 |
|  | T5 | 0.095 | -0.416,0.607 | 0.714 | 0.046 | -0.469,0.562 | 0.860 | 0.081 | -0.443,0.605 | 0.793 | 0.002 | -0.522,0.525 | 0.995 |
| Construction | T2 | -0.069 | -0.572,0.435 | 0.789 | -0.073 | -0.577,0.430 | 0.776 | -0.111 | -0.616,0.395 | 0.668 | -0.118 | -0.627,0.392 | 0.651 |
|  | T3 | 0.242 | -0.378,0.862 | 0.444 | 0.255 | -0.368,0.878 | 0.422 | 0.205 | -0.421,0.832 | 0.6520 | 0.163 | -0.475,0.800 | 0.617 |
|  | T4 | -0.350 | -0.913,0.213 | 0.223 | -0.264 | -0.877,0.350 | 0.399 | -0.275 | -0.899,0.350 | 0.389 | -0.336 | -0.967,0.295 | 0.297 |
|  | T5 | 0.246 | -0.339,0.831 | 0.410 | 0.256 | -0.330,0.842 | 0.392 | 0.272 | -0.334,0.877 | 0.379 | 0.247 | -0.360,0.854 | 0.426 |
| Healthcare | T2 | -0.052 | -0.817,0.712 | 0.894 | -0.095 | -0.870,0.678 | 0.808 | -0.101 | -0.880,0.677 | 0.799 | -0.155 | -0.928,0.619 | 0.695 |
|  | T3 | -0.442 | -1.305,0.420 | 0.315 | -0.398 | -1.278,0.482 | 0.376 | -0.392 | -1.277,0.492 | 0.385 | -0.438 | -1.317,0.442 | 0.330 |
|  | T4 | -0.395 | -1.218,0.428 | 0.347 | -0.444 | -1.277,0.339 | 0.296 | -0.509 | -1.351,0.334 | 0.237 | -0.519 | -1.371,0.333 | 0.232 |
|  | T5 | -0.593 | -1.525,0.340 | 0.213 | -0.675 | -1.618,0.269 | 0.161 | -0.745 | -1.699,0.208 | 0.125 | -0.853 | -1.814,0.107 | 0.082 |

T2: 6 months, T3: 12 months, T4: 18 months, T5: 24 months; Observations: total observations included in models for total/construction/healthcare; P-values ≤ 0.05 in bold.

Dependent variable: Change in pain between T1 and TX. Independent variable: Model 1: Static standing at work (minutes per work day); Model 2: As model 1 + Age, Gender, Smoking, BMI; Model 3: As model 2 + Heavy lifting, Kneeling; Model 3: As model 1 + Social climate, Decision control, Fair leadership, Empowering leadership.

**Supplementary B. Associations between total duration of dynamic standing at work (per 100 minutes) and change in LEPi during follow-up (Approach 2).**

|  |  | Model 1 | | | Model 2 | | | Model 3 | | | Model 4 | | |
| --- | --- | --- | --- | --- | --- | --- | --- | --- | --- | --- | --- | --- | --- |
|  |  | Observations = 426/215/211 | | | Observations = 418/214/204 | | | Observations = 417/213/204 | | | Observations = 412/212/200 | | |
|  |  | Coef. | 95% CI | P-value | Coef. | 95% CI | P-value | Coef. | 95% CI | P-value | Coef. | 95% CI | P-value |
| Total | T2 | 0.015 | -0.276,0.306 | 0.920 | 0.015 | -0.279,0.309 | 0.919 | 0.012 | -0.283,0.307 | 0.936 | 0.003 | -0.290,0.295 | 0.987 |
|  | T3 | 0.084 | -0.237,0.404 | 0.609 | 0.111 | -0.217,0.438 | 0.507 | 0.108 | -0.222,0.437 | 0.521 | 0.093 | -0.237,0.423 | 0.580 |
|  | T4 | -0.044 | -0.452,0.174 | 0.383 | -0.118 | -0.440,0.203 | 0.471 | -0.114 | -0.438,0.210 | 0.490 | -0.127 | -0.449,0.195 | 0.440 |
|  | T5 | 0.142 | -0.193,0.485 | 0.399 | 0.113 | -0.229,0.456 | 0.516 | 0.130 | -0.216,0.475 | 0.462 | 0.081 | -0.266,0.427 | 0.648 |
| Construction | T2 | -0.050 | -0.383,0.282 | 0.766 | -0.055 | -0.387,0.278 | 0.748 | -0.078 | -0.412,0.255 | 0.646 | -0.089 | -0.426,0.248 | 0.604 |
|  | T3 | 0.187 | -0.190,0.564 | 0.331 | 0.188 | -0.189,0.566 | 0.327 | 0.167 | -0.212,0.456 | 0.388 | 0.137 | -0.252,0.525 | 0.490 |
|  | T4 | -0.126 | -0.483,0.231 | 0.490 | -0.087 | -0.454,0.279 | 0.640 | -0.084 | -0.456,0.286 | 0.654 | -0.119 | -0.496,0.257 | 0.535 |
|  | T5 | 0.136 | -0.243,0.515 | 0.482 | 0.137 | -0.242,0.515 | 0.480 | 0.147 | -0.238,0.531 | 0.454 | 0.138 | -0.248,0.523 | 0.485 |
| Healthcare | T2 | 0.077 | -0.478,0.632 | 0.786 | 0.058 | -0.510,0.627 | 0.840 | 0.056 | -0.516,0.629 | 0.847 | 0.026 | -0.543,0.596 | 0.928 |
|  | T3 | -0.284 | -0.924,0.357 | 0.385 | -0.245 | -0.909,0.420 | 0.470 | -0.235 | -0.904,0.434 | 0.491 | -0.251 | -0.913,0.411 | 0.458 |
|  | T4 | -0.229 | -0.835,0.376 | 0.458 | -0.222 | -0.839,0.396 | 0.482 | -0.263 | -0.888,0.361 | 0.409 | -0.259 | -0.888,0.371 | 0.420 |
|  | T5 | -0.323 | -1.039,0.393 | 0.377 | -0.393 | -1.112,0.336 | 0.291 | -0.454 | -1.192,0.284 | 0.229 | -0.544 | -1.292,0.204 | 0.154 |

T2: 6 months, T3: 12 months, T4: 18 months, T5: 24 months; Observations: total observations included in models for total/construction/healthcare; P-values ≤ 0.05 in bold.

Dependent variable: Change in pain between T1 and TX. Independent variable: Model 1: Dynamic standing at work (minutes per work day); Model 2: As model 1 + Age, Gender, Smoking, BMI; Model 3: As model 2 + Heavy lifting, Kneeling; Model 3: As model 1 + Social climate, Decision control, Fair leadership, Empowering leadership.

**Supplementary C. Associations between maximum bouts of static standing at work (per 10 minutes) and change in LEPi during follow-up (Approach 2).**

|  |  | Model 1 | | | Model 2 | | | Model 3 | | | Model 4 | | |
| --- | --- | --- | --- | --- | --- | --- | --- | --- | --- | --- | --- | --- | --- |
|  |  | Observations = 426/215/211 | | | Observations = 418/214/204 | | | Observations = 417/213/204 | | | Observations = 412/212/200 | | |
|  |  | Coef. | 95% CI | P-value | Coef. | 95% CI | P-value | Coef. | 95% CI | P-value | Coef. | 95% CI | P-value |
| Total | T2 | -0.253 | -1.005,0.498 | 0.508 | -0.232 | -0.986,0.521 | 0.546 | -0.216 | -0.974,0.542 | 0.577 | -0.232 | -0.990,0.527 | 0.550 |
|  | T3 | -0.093 | -0.929,0.743 | 0.828 | -0.112 | -0.953,0.729 | 0.794 | -0.107 | -0.951,0.736 | 0.803 | -0.158 | -1.025,0.687 | 0.714 |
|  | T4 | -0.307 | -1.141,0.527 | 0.470 | -0.373 | -1.210,0.464 | 0.382 | -0.358 | -1.198,0.483 | 0.404 | -0.455 | -1.359,0.426 | 0.311 |
|  | T5 | -0.150 | -0.996,0.697 | 0.729 | -0.249 | -1.099,0.600 | 0.565 | -0.195 | -1.060,0.669 | 0.658 | -0.171 | -1.031,0.689 | 0.697 |
| Construction | T2 | -0.067 | -1.063,0.929 | 0.895 | -0.103 | -1.096,0.890 | 0.839 | -0.111 | -1.105,0.883 | 0.827 | -0.057 | -1.054,0.940 | 0.911 |
|  | T3 | 0.361 | -0.692,1.415 | 0.501 | 0.347 | -0.704,1.398 | 0.517 | 0.356 | -0.698,1.410 | 0.508 | 0.341 | -0.714,1.397 | 0.526 |
|  | T4 | 0.415 | -0.618,1.448 | 0.431 | 0.371 | -0.660,1.404 | 0.480 | 0.418 | -0.619,1.455 | 0.429 | 0.372 | -0.774,1.518 | 0.525 |
|  | T5 | 0.291 | -0.724,1.306 | 0.574 | 0.316 | -0.699,1.330 | 0.542 | 0.392 | -0.653,1.437 | 0.462 | 0.426 | -0.623,1.476 | 0.426 |
| Healthcare | T2 | -0.437 | -1.534,0.661 | 0.436 | -0.405 | -1.516,0.705 | 0.474 | -0.485 | -1.614,0.643 | 0.399 | -0.509 | -1.656,0.637 | 0.384 |
|  | T3 | -0.621 | -1.897,0.654 | 0.340 | -0.657 | -1.956,0.642 | 0.322 | -0.738 | -2.047,0.571 | 0.269 | -0.817 | -2.150,0.515 | 0.229 |
|  | T4 | -1.058 | -2.355,0.240 | 0.110 | -1.164 | -2.478,0.149 | 0.082 | -1.281 | -2.611,0.048 | 0.059 | -1.292 | -2.641,0.056 | 0.060 |
|  | T5 | -0.876 | -2.242,0.489 | 0.208 | -1.066 | -2.449,0.318 | 0.131 | -1.099 | -2.290,0.293 | 0.122 | -1.101 | -2.497,0.295 | 0.122 |

T2: 6 months, T3: 12 months, T4: 18 months, T5: 24 months; Observations: total observations included in models for total/construction/healthcare; P-values ≤ 0.05 in bold.

Dependent variable: Change in pain between T1 and TX. Independent variable: Model 1: Maximum bout of static standing at work (minutes per work day); Model 2: As model 1 + Age, Gender, Smoking, BMI; Model 3: As model 2 + Heavy lifting, Kneeling; Model 4: As model 3 + Social climate, Decision control, Fair leadership, Empowering leadership.

**Supplementary D. Associations between maximum bouts of dynamic standing at work (per 10 minutes) and change in LEPi during follow-up (Approach 2).**

|  |  | Model 1 | | | Model 2 | | | Model 3 | | | Model 4 | | |
| --- | --- | --- | --- | --- | --- | --- | --- | --- | --- | --- | --- | --- | --- |
|  |  | Observations = 426/215/211 | | | Observations = 418/214/204 | | | Observations = 417/213/204 | | | Observations = 412/212/200 | | |
|  |  | Coef. | 95% CI | P-value | Coef. | 95% CI | P-value | Coef. | 95% CI | P-value | Coef. | 95% CI | P-value |
| Total | T2 | 0.049 | -0.352,0.450 | 0.810 | 0.036 | -0.362,0.435 | 0.858 | 0.038 | -0.362,0.438 | 0.853 | 0.018 | -0.379,0.415 | 0.929 |
|  | T3 | -0.099 | -0.648,0.451 | 0.725 | -0.089 | -0.639,0.461 | 0.751 | -0.085 | -0.638,0.467 | 0.762 | -0.121 | -0.672,0.429 | 0.666 |
|  | T4 | -0.094 | -0.586,0.399 | 0.710 | -0.128 | -0.622,0.367 | 0.613 | -0.127 | -0.625,0.371 | 0.617 | -0.168 | -0.667,0.332 | 0.510 |
|  | T5 | -0.274 | -0.856,0.309 | 0.357 | -0.348 | -0.934,0.238 | 0.244 | -0.338 | -0.928,0.251 | 0.261 | -0.354 | -0.941,0.232 | 0.236 |
| Construction | T2 | 0.236 | -0.347,0.818 | 0.428 | 0.227 | -0.353,0.808 | 0.443 | 0.197 | -0.386,0.780 | 0.507 | 0.244 | -0.341,0.830 | 0.414 |
|  | T3 | 0.306 | -0.449,1.062 | 0.427 | 0.319 | -0.435,1.074 | 0.407 | 0.329 | -0.431,1.088 | 0.396 | 0.326 | -0.440,1.091 | 0.404 |
|  | T4 | -0.194 | -1.029,0.643 | 0.650 | -0.181 | -1.016,0.654 | 0.671 | -0.227 | -1.086,0.632 | 0.604 | -0.364 | -1.262,0.534 | 0.427 |
|  | T5 | -0.161 | -0.951,0.630 | 0.690 | -0.157 | -0.946,0.632 | 0.697 | -0.146 | -0.950,0.657 | 0.721 | -0.155 | -0.962,0.652 | 0.706 |
| Healthcare | T2 | -0.086 | -0.643,0.471 | 0.762 | -0.096 | -0.654,0.461 | 0.734 | -0.146 | -0.711,0.420 | 0.613 | -0.152 | -0.714,0.410 | 0.596 |
|  | T3 | -0.429 | -1.215,0.356 | 0.284 | -0.395 | -1.189,0.399 | 0.329 | -0.423 | -1.223,0.376 | 0.376 | -0.456 | -1.252,0.341 | 0.262 |
|  | T4 | -0.112 | -0.753,0.528 | 0.731 | -0.158 | -0.807,0.491 | 0.633 | -0.195 | -0.851,0.461 | 0.559 | -0.190 | -0.850,0.471 | 0.574 |
|  | T5 | -0.353 | -1.195,0.488 | 0.410 | -0.447 | -1.303,0.410 | 0.307 | -0.472 | -1.335,0.391 | 0.284 | -0.465 | -1.329,0.400 | 0.292 |

T2: 6 months, T3: 12 months, T4: 18 months, T5: 24 months; Observations: total observations included in models for total/construction/healthcare; P-values ≤ 0.05 in bold.

Dependent variable: Change in pain between T1 and TX. Independent variable: Model 1: Maximum bout of dynamic standing at work (minutes per work day); Model 2: As model 1 + Age, Gender, Smoking, BMI; Model 3: As model 2 + Heavy lifting, Kneeling; Model 4: As model 3 + Social climate, Decision control, Fair leadership, Empowering leadership.

**Supplementary E. Associations between total duration of on-feet activity (per 100 minutes) and average LEPi during follow-up (Approach 1).**

|  | Model 1 | | | Model 2 | | | Model 3 | | | Model 4 | | |
| --- | --- | --- | --- | --- | --- | --- | --- | --- | --- | --- | --- | --- |
|  | Observations = 303/154/149 | | | Observations = 297/153/144 | | | Observations = 296/152/144 | | | Observations = 293/151/142 | | |
|  | Coef. | 95% CI | P-value | Coef. | 95% CI | P-value | Coef. | 95% CI | P-value | Coef. | 95% CI | P-value |
| All workers | 0.269 | 0.011,0.528 | **0.041** | 0.351 | 0.069,0.634 | **0.015** | 0.318 | 0.022,0.614 | **0.035** | 0.304 | 0.005,0.603 | **0.047** |
| Construction | 0.288 | -0.012,0.589 | 0.060 | 0.297 | -0.006,0.601 | 0.055 | 0.294 | -0.036,0.624 | 0.080 | 0.172 | -0.189,0.533 | 0.350 |
| Healthcare | 0.496 | -0.054,1.045 | 0.077 | 0.491 | -0.095,1.076 | 0.100 | 0.443 | -0.129,1.015 | 0.129 | 0.335 | -0.265,0.934 | 0.274 |

Observations: total observations included in linear mixed models for all workers/construction/healthcare.

Dependent variable: the average pain intensity from 6 months to 24 months follow-up (T2, T3, T4, T5).

Independent variables:

Model 1: On-feet activity (dynamic standing + walking) (minutes per workday)

Model 2: As model 1 + Age, Gender, Smoking, BMI

Model 3: As model 2 + Heavy lifting, Kneeling

Model 4: As model 3 + Social climate, Decision control, Fair leadership, Empowering leadership

**Supplementary F. Associations between total duration of on-feet activity at work (per 100 minutes) and change in LEPi during follow-up (Approach 2).**

|  |  | Model 1 | | | Model 2 | | | Model 3 | | | Model 4 | | |
| --- | --- | --- | --- | --- | --- | --- | --- | --- | --- | --- | --- | --- | --- |
|  |  | Observations = 426/215/211 | | | Observations = 418/214/204 | | | Observations = 417/213/204 | | | Observations = 412/212/200 | | |
|  |  | Coef. | 95% CI | P-value | Coef. | 95% CI | P-value | Coef. | 95% CI | P-value | Coef. | 95% CI | P-value |
| Total | T2 | 0.014 | -0.221,0.248 | 0.908 | 0.015 | -0.222,0.251 | 0.903 | 0.013 | -0.225,0.251 | 0.915 | 0.008 | -0.228,0.243 | 0.950 |
|  | T3 | 0.092 | -0.163,0.347 | 0.479 | 0.116 | -0.145,0.378 | 0.383 | 0.113 | -0.150,0.376 | 0.398 | 0.103 | -0.160,0.365 | 0.444 |
|  | T4 | -0.074 | -0.323,0.177 | 0.564 | -0.055 | -0.312,0.201 | 0.674 | -0.051 | -0.309,0.207 | 0.697 | -0.061 | -0.319,0.196 | 0.640 |
|  | T5 | 0.150 | -0.116,0.417 | 0.268 | 0.126 | -0.143,0.395 | 0.358 | 0.138 | -0.133,0.410 | 0.318 | 0.109 | -0.164,0.381 | 0.434 |
| Construction | T2 | -0.036 | -0.300,0.228 | 0.789 | -0.039 | -0.303,0.255 | 0.770 | -0.054 | -0.319,0.211 | 0.692 | -0.060 | -0.327,0.207 | 0.661 |
|  | T3 | 0.183 | -0.113,0.478 | 0.225 | 0.183 | -0.112,0.478 | 0.224 | 0.164 | -0.133,0.461 | 0.278 | 0.142 | -0.163,0.446 | 0.361 |
|  | T4 | -0.050 | -0.330,0.230 | 0.726 | -0.024 | -0.309,0.261 | 0.870 | -0.018 | -0.307,0.271 | 0.902 | -0.042 | -0.335,0.251 | 0.777 |
|  | T5 | 0.121 | -0.174,0.415 | 0.422 | 0.116 | -0.178,0.410 | 0.440 | 0.130 | -0.169,0.428 | 0.394 | 0.124 | -0.175,0.424 | 0.416 |
| Healthcare | T2 | 0.073 | -0.409,0.55 | 0.766 | 0.054 | -0.442,0.549 | 0.832 | 0.056 | -0.444,0.556 | 0.827 | 0.025 | -0.475,0.524 | 0.923 |
|  | T3 | -0.271 | -0.829,0.287 | 0.341 | -0.237 | -0.824,0.350 | 0.429 | -0.227 | -0.818,0.364 | 0.452 | -0.239 | -0.825,0.347 | 0.424 |
|  | T4 | -0.178 | -0.707,0.350 | 0.508 | -0.159 | -0.702,0.383 | 0.565 | -0.194 | -0.743,0.355 | 0.488 | -0.193 | -0.748,0.363 | 0.497 |
|  | T5 | -0.253 | -0.882,0.376 | 0.431 | -0.320 | -0.963,0.324 | 0.330 | -0.371 | -1.022,0.280 | 0.264 | -0.424 | -1.086,0.237 | 0.208 |

T2: 6 months, T3: 12 months, T4: 18 months, T5: 24 months; Observations: total observations included in models for total/construction/healthcare; P-values ≤ 0.05 in bold.

Dependent variable: Change in pain between T1 and TX. Independent variable: Model 1: On-feet activity (dynamic standing + walking) at work (minutes per work day); Model 2: As model 1 + Age, Gender, Smoking, BMI; Model 3: As model 2 + Heavy lifting, Kneeling; Model 3: As model 1 + Social climate, Decision control, Fair leadership, Empowering leadership.
